# Traditional Chinese medicine inhibits PD-1/PD-L1 axis to sensitize cancer immunotherapy: a literature review

**DOI:** 10.3389/fonc.2023.1168226

**Published:** 2023-06-16

**Authors:** Huilan Zheng, Gang Wang, Ming Liu, Hongbin Cheng

**Affiliations:** ^1^Department of Dermatology, Hospital of Chengdu University of Traditional Chinese Medicine, Chengdu University of Traditional Chinese Medicine, Chengdu, Sichuan, China; ^2^National Engineering Research Center for Biomaterials, Sichuan University, Chengdu, Sichuan, China; ^3^Department of Medical Oncology/Gastric Cancer Center, West China Hospital, Sichuan University, Chengdu, Sichuan, China

**Keywords:** traditional Chinese medicine, cancer immunotherapy, sensitization, immune checkpoint inhibitor, PD-1/PD-L1 axis

## Abstract

The Programmed death-1 (PD-1) and its programmed death-ligand 1 (PD-L1) comprise the PD-1/PD-L1 axis and maintain tumor immune evasion. Cancer immunotherapy based on anti-PD-1/PD-L1 antibodies is the most promising anti-tumor treatment available but is currently facing the thorny problem of unsatisfactory outcomes. Traditional Chinese Medicine (TCM), with its rich heritage of Chinese medicine monomers, herbal formulas, and physical therapies like acupuncture, moxibustion, and catgut implantation, is a multi-component and multi-target system of medicine known for enhancing immunity and preventing the spread of disease. TCM is often used as an adjuvant therapy for cancer in clinical practices, and recent studies have demonstrated the synergistic effects of combining TCM with cancer immunotherapy. In this review, we examined the PD-1/PD-L1 axis and its role in tumor immune escape while exploring how TCM therapies can modulate the PD-1/PD-L1 axis to improve the efficacy of cancer immunotherapy. Our findings suggest that TCM therapy can enhance cancer immunotherapy by reducing the expression of PD-1 and PD-L1, regulating T-cell function, improving the tumor immune microenvironment, and regulating intestinal flora. We hope this review may serve as a valuable resource for future studies on the sensitization of immune checkpoint inhibitors (ICIs) therapy.

## Introduction

Cancer immunotherapy is an immunological principles-based treatment to enhance the host’s immune response against tumor cells, with the ultimate goal of tumor clearance (1, 2). As a revolutionary advancement in tumor treatment after surgery, radiation, and chemotherapy, it has the benefits of good efficacy, broad indications, long-lasting remission, and high targetability (3, 4). One of the most intriguing and efficient methods for enabling the body’s immune cells to eradicate tumors is immune checkpoint blockage. An emerging trend in oncology (5, 6) is next-generation combination therapies that simultaneously use several pathways to enhance tumor immunosuppression and provide an anti-tumor effect. However, drug toxicity, drug resistance, and immune-related adverse effects are only a few obstacles faced when using cancer immunotherapy, whether as a single agent or in combination (7–9). Therefore, we focused on the existing cancer immunotherapy sensitization (10).

Traditional Chinese medicine, including monomers, Chinese herbal formulas, acupuncture, moxibustion, and other types, is often used as adjuvant therapy for cancer patients. As a result of its ability to directly inhibit or kill tumor cells (11) or to decrease tumor immune escape through innate and adaptive immunity modulation, it shows considerable potential as a cancer immunotherapy (12, 13). Several recent *in vivo* and *in vitro* investigations have shown that TCM may increase the therapeutic sensitivity of cancer immunotherapy and that TCM, in conjunction with PD-1/PD-L1 monoclonal antibodies, can succeed even more. This review aimed to provide an understanding of the potential sensitization mechanisms of PD-1/PD-L1 axis-based TCM therapy and the synergistic effects of cancer immunotherapy.

## PD-1/PD-L1 axis

First identified in 1992 by Tasuku Honjo (14), Programmed death-1 (PD-1) is a transmembrane glycoprotein with a molecular weight of 50-55 kDa (15) expressed on activated T cells, B cells, and macrophages. Under normal body conditions, it helps immune cells distinguish between healthy and damaged tissue, hence maintaining peripheral immune tolerance. In addition to its involvement in regulating autoimmunity, cancer immunity, and infection immunity, it also significantly regulates other types of immune responses (16).

The term “PD-1/PD-L1 axis”, often mentioned in cancer immunity, refers to the signaling pathway in which PD-1 and its PD-L1 ligand are the primary molecules (17). PD-L1, also known as CD274 or B7-H1, can be expressed on various immune cells such as T cells, B cells, NK cells, macrophages, dendritic cells, and epithelial cells, and can also exist in an extracellular form as exosomes or soluble proteins (18, 19). In pathological conditions, PD-L1 can be overexpressed on the surface of multiple tumor cells, leading to tumor invasion (20). Immune checkpoints PD-1 and PD-L1 have been the subject of numerous investigations, and PD-1/PD-L1 blockade-based immunotherapy has had unprecedented success. Additionally, PD-L2, a less well-studied PD-1 ligand that can be expressed independently of PD-L1 in tumor tissue (21), is mainly found on antigen-presenting cells and inhibits CD4+ T cell activation and proliferation upon binding to PD-1 (22).

## Tumor immune escape and PD-1/PD-L1 axis

Cancer immunity is a cyclical process that begins with releasing cancer cell antigens and their presentation to T lymphocytes by antigen-presenting cells. Naive T cells then attach to MHC class I antigen fragments and differentiate into CD8+ T cells, which are cytotoxic effector T cells (23). The primed and activated effector T cells gradually migrate and infiltrate into the tumor tissues, identify the tumor by specific T cell receptor (TCR) and antigen binding, and then release perforin, granzyme B (GZMB), and interferon-gamma (IFN-γ) to induce killing of tumor cells (24). Tumor immune escape can result from ineffectiveness at any stage of the cancer immune cycle, including loss of antigenic epitopes, decreased antigen presentation, unsuccessful activation of tumor-specific T cells, and inadequate infiltration of effector T cells (25). Due to a chronic inflammatory environment and constant exposure to tumor antigen stimulation (26, 27), CD8+ T cells — the primary driver of anti-tumor immunity — might gradually become dysfunctional or exhausted. The PD-1/PD-L1 axis, on the other hand, may aggravate the induction of T cell apoptosis, anergy, and exhaustion, hence contributing to primary tumor immune escape and poor cancer prognosis (28–30). PD-1 destroys T cell functions by dephosphorylating molecules downstream of the TCR by recruiting Src homology 2-containing protein tyrosine phosphatase 2 (SHP2) (31).

Tumor immune escape occurs in the complex tumor immune microenvironment (TME), which is the site of antagonism between immune effects and immune suppression. The natural killer (NK) cells, macrophages, T helper 1 (Th1) cells, and Th17 cells, differentiated from CD4+ T cells, positively promote tumor immunity (32–34). In contrast, regulatory T (Treg) cells, tumor-associated macrophages (TAMs), and myeloid-derived suppressor cells (MDSCs) mainly act as negative characters and together promote tumor immune escape and tumor metastasis (35, 36). These cells can then release cytokines that regulate the PD-1/PD-L1 axis directly, and most of them can activate the PD-1/PD-L1 axis, producing a superimposed suppressive effect on tumor immunity (37). Interferon-γ (IFN-γ), produced by activated T cells, can boost the immune system’s capacity to kill tumors in the preprophase. It may also up-regulate PD-L1 expression in tumor cells by activating the JAK/STAT1/IRF1 signaling pathway (38, 39). PD-L1 is up-regulated in tumor cells when TNF-α activates the NF-κB signaling pathway, increasing expression of the IFN-γ receptor (IFNGR1 and IFNGR2) (40). Through activation of NF-κB and ERK1/2 signaling pathways in tumor cells, interleukin-17(IL-17) released by Th17 cells may up-regulate PD-L1 expression (41). The transcription factor STAT3 is responsible for the effects of macrophage-derived IL-6 on CD8+ T cells (42). Infiltrating TAM may cause PD-L1 expression by secreting IL-6 and TNF-α (43). However, PD-1 expression in T cells may also be up-regulated by common gamma-chain cytokines, such as IL-2, IL-7, IL-15, and IL-21 (44). The PD-1/PD-L1 axis may also regulate cytokines in the TME. Transforming growth factor-β (TGF-β) has been shown to inhibit T-cell-induced immunological infiltration by stimulating collagen production to form a physical barrier (45). However, PD-L1 has increased tumor cell proliferation and invasion by up-regulating BAG-1 expression, lowering TGF-β levels, and subsequently down-regulating SMAD4 (46).

## Cancer Immunotherapy and the sensitization strategy of TCM

Cancer immunotherapy can be divided into two categories based on the host immune system’s role in fighting tumors: active and passive immunotherapy (47). Active immunotherapy utilizes tumor antigens to increase the tumor’s immunogenicity and directly stimulate an immune response (48). This includes various therapeutic cancer vaccines. On the other hand, passive immunotherapies, such as monoclonal antibodies, adoptive cell therapy, and oncolytic virotherapy (49), rely on pre-induced antibodies or immune cells to mediate the host immune system’s response against the tumor cells (50). It has been suggested that passive immunotherapies may also possess moderate active immunological activity (51). Immune checkpoint inhibitors (ICIs) therapy targeting the PD-1/PD-L1 axis has been developed to address this (52, 53). This therapy utilizes monoclonal antibodies against PD-1 and PD-L1 to bind to tumor cells and T cells and overcome immunosuppression to enable effector functions. When tumor cells utilize the PD-1/PD-L1 axis to prevent an immunological attack, cancer immunotherapy using PD-1/PD-L1 inhibition can help the immune system re-identify and eliminate cancerous cells. Anti-PD-1 antibodies such as pembrolizumab and nivolumab, as well as anti-PD-L1 antibodies like atezolizumab and durvalumab, have been approved for the treatment of advanced melanoma, non-small cell lung cancer (NSCLC), renal cell carcinoma (RCC), and other cancers making them the foremost therapeutic agents in the oncology field (54). However, despite being the leading therapeutic agent in oncology, the current population that benefits from ICIs therapy falls short of expectations. This has prompted a search for more sensitization techniques to maximize anti-cancer effectiveness. Existing sensitization methods aim to directly impact low-antigen and effector T cells or indirectly influence tumor growth by inhibiting angiogenesis and mesenchymal transition (10, 55). Studies on the sensitization of this axis should receive adequate attention considering the importance of the PD-1/PD-L1 axis in tumor immunosuppression.

Based on the fundamental principles of Chinese medicine, TCM treatment seeks to restore a healthy Yin-Yang equilibrium inside the body. “Yin” denotes all adverse, inhibitive bodily processes, whereas “Yang” denotes all advantageous, stimulating processes. This action can be regarded as Yin when the PD-1/PD-L1 axis is activated and further inhibits the organism’s immune system. The following four diagnostic techniques (“Si Zhen” in Chinese) are used by TCM practitioners to obtain data: 1) Inspection (“Wang Zhen”) refers to the doctors’ ability to examine the patient’s bodily and mental conditions, especially the tongue. 2) Auscultation and olfaction (“Wen Zhen”) refer to the medical term for the process by which a doctor makes a diagnosis by hearing and smelling the patient’s speech (including breathing, coughing, belching, etc.) and the smell of feces to conclude. 3) Inquiry (Wen Zhen) refers to the TCM physician’s use of conversation to provoke information from the patient and others about the disease’s onset, progression, present-day symptoms, and any prior treatments. 4) Pulse-feeling and palpation (“Qie Zhen”) necessitate the medical professional to feel the patient’s pulse as well as their skin, chest, and abdomen to look for any abnormal symptoms. The patient’s state can be determined by these four criteria, which can assist in selecting an optimal personalized TCM treatment and increase the efficacy of cancer immunotherapy. The disease will be treated with Chinese medicines and physical procedures once the relevant therapeutic parameters have been determined through the empirical debate. Herbal monomers have been the research subject for their potential in herbal medicine. Natural products like polysaccharides, flavonoids, alkaloids, saponins, and organic acids may be obtained from either animal or plant extracts and are considered endogenous active ingredients. Various herbal remedies may share the same active ingredient (56).

**Figure 1:**
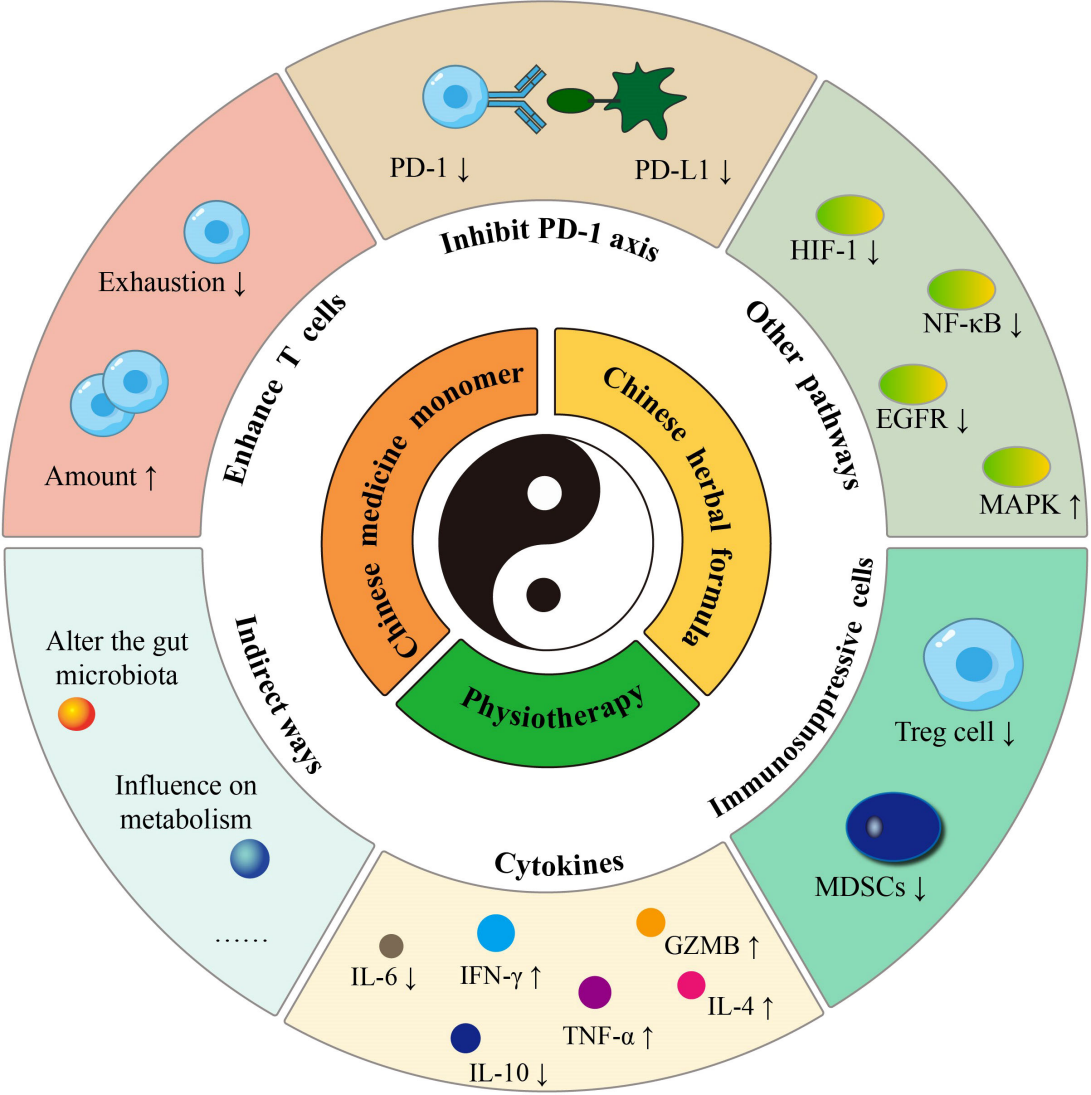
The sensitization strategy of TCM therapy.

Consequently, a single herbal medicine often contains numerous active substances and exhibits multi-component, multi-target, and multi-pathway characteristics. Herbal monomers have been shown in pharmacological studies to have anti-inflammatory, antibacterial, antiviral, antioxidant, anti-tumor, and immune system-modulating activities (57). Several herbs with different therapeutic effects are combined into a single Chinese herbal formula according to precise combination criteria. (a) Sovereign (the principal medicinal herb for the major signs), (b) Minister (the herb that assists the sovereign), (c) Assistant (the herb that acts as an adjuvant or restraining agent), and (d) Guide (the herb that can harmonize all medicinal properties and induce them to specific meridians) are the four standard components of each procedure. As a result, herbs that kill tumors, modulate the immune system and mitigate immunotherapy’s adverse effects are frequently combined in a single formula to assist cancer immunotherapy. It may be broken down further into tonifying methods, pathogen elimination approach, and both based on the general guidelines. According to a case report study, Chinese herbal formula successfully treated cases of immune-related lichenoid dermatitis caused by sintilimab treatment in NSCLS patients (58). Acupuncture, moxibustion, cupping, and other methods from TCM are also often employed as adjuvant therapies for cancer patients. These physiotherapies primarily have anti-tumor and immune-boosting benefits by stimulating the acupuncture points of meridians and collaterals. The TCM-sensitized tumor immunotherapy includes killing tumor cells and enhancing T cell function, inhibiting the PD-1/PD-L1 axis, and improving the tumor immunosuppressive microenvironment to exert a sensitizing effect (**Figure 1** by Adobe Illustrator). The latter can be manifested in promoting tumor-killing cells, suppressing immunosuppressive cells, and regulating cytokine secretion. Several animal investigations of TCM therapies in tumor immunotherapy sensitization have been carried out, and clinical trials with future translation studies are anticipated to be carried out subsequently.

## Chinese medicine monomers sensitize cancer immunotherapy

### Polysaccharides

Shortness of breath, exhaustion, palpitations, and thirst are all signs of qi deficit, and ginseng is an effective tonic for restoring vital energy. According to studies, ginseng polysaccharides (GPs), a key component of *Panax ginseng*, have been shown to reduce inflammation, boost adaptive immunity, and enhance gut flora metabolism (59–61). Research by Jumin Huang et al. (62) showed that GPs could positively impact cancer immunotherapy, both alone and in combination with the αPD-1 monoclonal antibody (αPD-1mAb). In Lewis lung cancer mice, GPs alone inhibited tumor growth and reduced tumor volume and weight. The combination of GPs and αPD-1mAb had an even more substantial effect, resulting in 65.1% tumor growth suppression than αPD-1mAb alone. This combined treatment also increased the CD8+/CD4+ T cells’ ratio. It enhanced the release of immune effector cytokines (IFN-γ, TNF-α, GZMB) in both peripheral and tumor tissue and suppressed Treg cells to improve tumor immunosuppression. Furthermore, GPs improved gut microbiota and further affected tryptophan metabolism through gut microbes. It also converted the immune response phenotype of the tumor, restoring response to αPD-1mAb in mice transplanted with fecal samples from PD-1 non-responders, thereby expanding the potential for PD-1 blockade therapy.

In addition to treating edema, perspiration, and diarrhea, *Attractylodes macrocephala* strengthens the spleen and improves Qi. The Polysaccharide of Atractylodis Macrocephalae Rhizoma (PAMR) could function as an immune adjuvant that can activate macrophages through NF-κB and JAK/STAT signaling pathways (63, 64). Yicun Han et al. (65) found that PAMR could inhibit the proliferation of PD-L1 high-expressing esophageal cancer cells *in vitro* and reduce the expression of PD-L1. MicroRNA-34a (miR-34a), on the other hand, could effectively inhibit PD-L1 expression (66). By performing silencing and overexpression assays on miR-34, this study discovered that PAMR inhibited PD-L1 by increasing miR-34a levels. Regarding the regulatory mechanism, microRNAs are a class of small noncoding RNAs that can control gene expression by degrading mRNA or inhibiting translation. Various microRNAs have been shown to exert anti-tumor effects in tumors; therefore, studying Chinese herbal medicine to sensitize cancer immunotherapy from this perspective is a good idea.

*Astragalus* may relieve chronic ulcers, indigestion, coughing, and sweating by mobilizing the spleen, stomach, qi, and blood. Astragalus polysaccharides (APS), a vital component of the *Astragalus*, have been found to possess anti-inflammatory, antiviral, immune-enhancing, and gut-microbiome-regulating properties (67, 68). According to a study by Guiqing Ding et al. (69), treatment with APS significantly reduced the size of melanoma tumors in mice after just 14 days compared to the model group. Analysis of the TME showed that APS increased the number of CD8+ T cells and dendritic cells while reducing the proportion of immunosuppressive MDSCs. Additionally, APS reduced the expression of cytokines such as IL-1β, IL-6, TGF-β, and arginase-1, making it easier for CD8+ T cells to target and attack the tumors. 16S rRNA next-generation sequencing results showed that APS could reshape the gut microbiome and regulate metabolism, enhancing the body’s anti-cancer immune response.

Furthermore, research by Xinrui Sha et al. (70) showed that honey-processed Astragalus polysaccharides (HP-APS) could directly inhibit the proliferation of A549, MC38, and B16 tumor cell lines and trigger apoptosis. It also supported the maturation and activation of dendritic cells, improving the presentation of tumor antigens. In melanoma mice, HP-APS treatment reduced the size of tumors, increased the proportion of CD8+ T cells, and resulted in a slower rate of tumor growth when combined with PD-1 monoclonal antibody (PD-1 mAb) treatment, indicating a possible synergistic effect.

### Alkaloids

*Tetradium ruticarpum* dissipates cold, increases Yang, and can be used to treat cold-related pain, vomiting, and diarrhea. Evodiamine, a new cancer-fighting alkaloid extracted from *Tetradium* fruit, has shown promise in treating prostate cancer (71, 72), hepatocarcinoma (73), and gastric cancer (74). Zebo Jiang et al. (75) found that Evodiamine directly stopped the growth of lung cancer cell lines (H1650 and H1975), causing them to undergo apoptosis and stopping their cell cycle in the G2 phase. This is due to its effects on the *MUC1-C* gene, triggering a cancer cell’s transition from epithelial to mesenchymal (EMT) and promoting cancer cell invasion (76). Evodiamine was observed to decrease the expression of PD-L1 mRNA and protein 24 hours later; it also held for IFN-induced PD-L1 cell membrane expression. In co-culture with peripheral blood mononuclear cells, Evodiamine reduced T-cell apoptosis and increased the IFN-γ and GZMB to enhance immune effector capability. In the Lewis lung cancer mouse model, Evodiamine inhibited tumor growth, improved mouse survival, and improved the abundance, viability, and effectiveness of CD8+ T cells. Furthermore, when combined with PD-1 mAb, the treatment resulted in more tumor-infiltrating CD8+ T cells and greater secretion of tumor-killing factors like IFN-γ, TNF-α, and GZMB.

*Sophora flavescens* is a herbal remedy that helps treat diarrhea, bloody stools, jaundice, dermatitis, and clear heat and moisture. Oxymatrine, a quinazine alkaloid extracted from it, has been shown to have antiviral properties in treating chronic hepatitis B (77). Cellular tests have been performed by S. Hua et al. (78). They observed that oxymatrine inhibited cell viability in colorectal cancer cells, and the inhibition became more pronounced with increasing concentrations. When IFN-γ stimulated PD-L1 production in tumor cells, oxymatrine dramatically decreased this stimulation by controlling DNA demethylation.

### Polyphenols

Clinical applications of the Chinese herb *Rhus chinensis Mill* include the treatment of coughing, bloody coughing, sweating, chronic diarrhea, and early ejaculation. Gallic acid (GA) is a polyphenolic compound derived by fermentation from *Rhus chinensis Mill* that can suppress tumor and tumor-induced oxidative damage (79). According to a study by Biaolong Deng et al. (80), GA negatively impacts the phosphorylation of STAT3 and the expression of the *Usp21* gene. The expression of the *Usp21* gene leads to the degradation of the Foxp3 protein, which is crucial for the immunosuppressive function of Treg cells and results in the formation of Th1-like Treg cells. Similarly, GA also deubiquitinates PD-L1 expression in Treg cells. In colon cancer, MC38 tumor-bearing mice, both GA alone and with anti-PD-1 antibodies, inhibited tumor growth and prolonged survival. The combination therapy inhibited Foxp3 and PD-L1 protein expression in Treg cells more effectively than the anti-PD-1 antibody alone. The combination therapy also improved the effect of CD8+ cytotoxic T cells by producing more IFN-γ, reducing T-cell exhaustion, core factor Thymocyte selection-associated high-mobility group box (TOX), and immune checkpoint LAG3, increasing TNF-α and delaying the progression of colorectal cancer.

A rhizome from the plant *Curcuma longa* L., turmeric contains blood-activating properties. It can treat bruises, dysmenorrhea, joint discomfort, and bruises, as well as hypochondriacal and chest pain. A polyphenol from turmeric called curcumin has immune-boosting, anti-cancer, anti-inflammatory, and antioxidant properties (81, 82). It has been suggested to reduce paclitaxel resistance in the treatment of cancer (83). Curcumin’s therapeutic advantages for head and neck squamous cell carcinoma (HNSCC) were studied by Lihua Liu et al. (84). Curcumin decreased PD-L1 and PD-L2 expression in HNSCC cell lines in an *in vitro* cellular experiment without negatively impacting healthy human fibroblast cells. When curcumin and PD-L1 antibody were combined, tumor development was considerably inhibited, and co-cultured CD8+ T cells released more IFN-γ and GZMB. Curcumin demonstrated a comparable inhibition on tumor growth in oral cancer model mice, and SCC15 cell-established HNSCC tumor-bearing animals, and PD-L1 and PD-L2 expression inhibition was also reported. Higher CD4+ and CD8+ T cells were observed in the mouse spleen and blood, indicating immune system activation and reduced Treg cell numbers.

### Flavonoids

*Psoralea corylifolia* L. is a kidney tonic that treats impotence, frequent urination, and back pain caused by insufficient kidney supply. Neobavaisoflavone (NBIF) is a crucial component of *Psoralea corylifolia* L. that has been shown to have chemosensitizing benefits for treating glioma (85). It also can inhibit STAT3 activation and suppress NSCLC cell proliferation (86). Jufeng Guo et al. (87) found that NBIF inhibited tumor growth in breast and small-cell lung cancer mice. In breast cancer 4T1 mice, it increased the number of CD4+ and CD8+ T cells in the TME and spleen. Moreover, it also supported the production of IFN-γ, perforin, GZMB, and Ki-67, which enhanced the immune effector function. In addition, the study found that NBIF reduced spleen MDSC activity by decreasing STAT3 phosphorylation and lowered intracellular arginase-1 and reactive oxygen species (ROS) levels, which unrestricted T cells. In combination therapy, NBIF enhanced anti-PD-1 efficacy in 4T1 tumors insensitive to anti-PD-1 treatment and improved the peripheral immune environment. It not only increased spleen IFN-γ+CD4+ T cells, IFN-γ+CD8+ T cells, and memory CD4+ and CD8+ T cells in the blood.

Numerous plants contain quercetin, also present in Chinese herbal remedies derived from radix bupleuri, mulberry leaf, hawthorn, etc. It possesses antioxidant, antimicrobial, and anti-inflammatory properties (88, 89). In a competitive enzyme-linked immunosorbent test developed by Lei Jing et al. (90), it was discovered that quercetin dihydrate could prevent 90% of the interaction between glycosylated PD-1 or PD-L1 in HEK293 cells. Quercetin dihydrate encouraged Jurkat T cells to secrete IL-2 in a co-culture system with a cell line with high PD-L1 expression. On the other hand, it killed tumor cells in unstimulated peripheral blood mononuclear cells. In tumor-bearing mice, quercetin dihydrate decreased tumor growth without affecting body weight, increasing CD8+ T cells and secreting more GZMB and IFN-γ.

### Lactones

The herb known as *Andrographis paniculata* is used to detoxify the body, clear heat, and treat various diseases such as colds, sore throats, lung abscesses, skin infections, etc. Andrographolide (AD) is a diterpene lactone compound found in the *Andrographis paniculata* (Burm.F.) Nees has been shown to have anti-cancer and antiviral properties. According to a study by Xuanrun Wang et al. (91), AD effectively inhibited the growth and promoted apoptosis of NSCLC cell lines (H1299, H1975). It works by binding to STAT3 and inhibiting its phosphorylation, significantly enhancing p62-dependent autophagy and reducing IFN-γ-induced PD-L1 expression. The study also found that AD increased levels of ROS in tumor cells, promoting oxidative stress and autophagy. However, this effect could be mitigated by the antioxidant N-acetyl-L-cysteine (NAC). In mice models with Lewis lung cancer and H1975 xenografts, AD prolonged the survival time and inhibited tumor growth without causing harm to vital organs. When combined with PD-1 mAb treatment, AD further reduced tumor volume and weight, suppressed PD-L1 expression, and positively regulated the TME. The treatment increased the number of CD8+ T cells infiltrating the tumor and increased levels of IFN-γ, GZMB, and TNF-α while decreasing the number of Treg cells in the tumor, blood, and spleen.

The root of *Inula helenium* L., known as Inulae Radix, is used to treat chest, stomach, and abdomen distension and pain, as well as gastrointestinal problems such as vomiting and diarrhea. Jaemoo Chun et al. (92) discovered that despite its moderate anti-tumor effect, a sesquiterpene lactone-rich fraction of I. helenium (SFIH) alone, substantially reduced tumor volume in MC38-bearing mice, when combined with PD-1 monoclonal antibody, and survival rate, increased significantly. Immunohistochemical analysis revealed that the combined treatment enhanced CD8+ T cell and macrophage infiltration increased GZMB secretion, and decreased TGF-β1 expression. While the combination treatment group demonstrated a decrease in MDSCs and an increase in M1-like macrophages, no significant effect on TAMs was observed for the duration of treatment. Furthermore, the study used RNA sequencing to discover that combined treatment enhanced apoptosis, interferon-gamma, and inflammatory responses.

## Herbal extracts sensitize cancer immunotherapy

*Platycodon grandiflorum* is a medicinal plant used primarily to treat pulmonary diseases. It contains active ingredients, such as Platycodin D (93), which has numerous pharmacological benefits, including anti-angiogenic, anti-tumor, and antioxidant properties (94, 95). Ruijie Yang et al. (96) conducted network pharmacology and HPLC tests and discovered that Platycodon D and Platycodon D3 were abundant in *Platycodon grandiflorum*. Further bioinformatics analysis showed that this herb had strong anti-tumor and immunomodulatory potential. *In vitro*, studies showed that PG (Platycodon D: Platycodon D3 = 1.5:1) significantly reduced the survival of several tumor cell lines (LLC, H1975, A549, CT26, and B16-F10), with LLC cell lines being the most sensitive. Using a mouse model of LLC-bearing C57BL/6 mice, PG was found to have significant inhibitory effects on tumor growth and prolonged lifespan. PG inhibited the phosphorylation of STAT3, down-regulated the expression of VEGF-A, and reduced the PD-1 membrane expression, leading to a decrease in PD-1+ CD8+ T cells in the TME. Additionally, PG promoted the proliferation of CD8+ T cells and increased the secretion of IFN-γ and GZMB to improve the killing effect on tumors. The study found that although PG alone had strong immunomodulatory and tumor-suppressive effects, raising tumor growth inhibition (TGI) to 57% at day 18 (compared to 36% with anti-PD-L1 antibodies), combination treatment did not show any appreciable improvement in survival time or reduction in tumor size.

Traditional Chinese herb *Taxus chinensis* var. *mairei*, formerly employed as an anthelmintic in ancient texts, has been discovered to have anti-tumor properties. Its aqueous extract (AETC) induced apoptosis in NSCLC cells via the Hippo-YAP pathway, which may drastically reduce tumor growth in tumor-bearing nude mice (97). Shuying Dai et al. (98) confirmed the standardization of the AETC by using HPLC assays to measure the levels of ginkgetin and quercetin. *In vitro*, studies showed that AETC decreased the survival of LLC and HCC827 lung cancer cells by breaking down CD47 through ubiquitination. CD47 on tumor cells interacts with the phagocytes’ SIRPα, sending a “do not eat me” signal and hindering the innate immune response (99). AETC was also found to enhance the phagocytic function of macrophages, which was demonstrated by the increased average phagocytic index. In a study with LLC-bearing mice, combining AETC with anti-PD-1 therapy resulted in a greater tumor size and weight reduction than monotherapy. It was accompanied by a lower level of PD-1 in macrophages and a smaller percentage of Treg cells, indicating a reversal of the tumor’s immune-suppressing microenvironment.

## Chinese herbal formulas sensitize cancer immunotherapy

### Eliminating pathogen method

The Gegen Qinlian decoction (GQD) is a formula from the “*Treatise On Cold Damage Disease*” book by the ancient Chinese physician Zhongjing Zhang. It is commonly used to treat ulcerative colitis (100) and acute gastroenteritis, and its purpose is to relieve external evil and remove internal heat, thereby restoring the balance between the exterior and interior. In a study by Ji Lv et al. (101), the combination of GQD with anti-PD-1 mAb therapy inhibited tumor growth in colorectal cancer CT26 tumor-bearing mice, with a maximum TGI value of 70.526% at day 32 (vs. 48.216% in the anti-PD-1 group). Furthermore, this study discovered that GQD maintained modulation of the gut microbiome and that the combination treatment modulated glycerophospholipid metabolism and sphingolipid metabolic pathways, thereby encouraging an indirect immune-enhancing effect. While PD-1 inhibition was more pronounced in the combination treatment group, it was also observed that the CD8+ T-cell ratio and the immune infiltration density were higher in TME in the combination treatment group, suggesting a beneficial synergistic effect.

Additionally, the study by Yihua Xu et al. (102) explored the anti-tumor effects of Dahuang Fuzi Baijiang decoction (DFB), another classic formula from Zhongjing Zhang, in treating colorectal cancer. The results showed that DFB effectively limited tumor growth in mice with MC38 tumors. In TME, DFB enhanced the infiltration of CD8+ T cells while suppressing their exhaustion and PD-1 expression. The treatment also reduced the IL-6 level in tumor tissues, but no synergistic effect was found with the combination therapy. Therefore, the suppression of terminal T cells by DFB helps to preserve the killing function of tumor immunity, making it a valuable addition to immune checkpoint blockade therapy.

Qingfei Jiedu decoction (QFJDD) is a formula that acts on the lung system, clears heat, and removes toxins. Junjie Pan et al. (103) found that serum containing QFJDD could downregulate PD-L1 expression in lung cancer A549 cells. Moreover, multiple signaling pathways can regulate the PD-1/PD-L1 axis, such as HIF-1, EGFR, NF-κB, and MAPK. Among them, hypoxia-inducible transcription factors (HIFs) are the primary molecules of the hypoxic phenotype in the TME. They can up-regulate PD-L1 levels by binding the hypoxia-response element in the PD-L1 proximal promoter (104, 105). The expression of PD-L1 can be directly induced by the epidermal growth factor receptor (EGFR) and mitogen-activated protein kinase (MAPK) (106, 107). At the same time, NF-κB is a downstream molecule of PD-1 that activates further by recruiting SHP2 through phosphorylation (108). Pan’s study found that QFJDD decreased the expression of critical genes involved in the HIF-1, EGFR, and NF-κB signaling pathways and increased the activity of the MAPK/AKT signaling pathway. In mice with Lewis lung cancer, the treatment also reduced PD-L1 levels in the tumor tissue and increased the proportion of CD8+PD-1+ T cells in the spleen, promoting the immune response.

### Tonifying method

The Buzhong Yiqi decoction (BYD) is an ancient Chinese herbal formula created by physician Dongyuan Li, used to treat digestive problems by promoting Yang energy and nourishing the spleen and stomach. It can be utilized in visceral prolapse, chronic gastroenteritis, muscle fatigue, and excessive menstruation. Ruihan Xu et al. (109) found that combining modified Buzhong Yiqi decoction (mBYD) with 5-fluorouracil prolonged survival time and improved thymus and spleen indexes in mice with gastric cancer. The combined treatment also increased CD4+/CD8+ T cells in peripheral blood, and a reduction in CD8+ PD-1+ T cells and PD-1+ Treg cells was observed. The study found that mBYD inhibits the PD-1/PD-L1 axis in tumors by downregulating the PI3K/AKT signaling pathway and directly promoting T cell proliferation and IFN-γ concentration to increase effector T cell toxicity. Clinical studies also confirmed the immunomodulatory abilities of mBYD, as chemotherapy combined with mBYD significantly reduced the proportion of CD8+ PD-1 + T cells.

Shu Yu Pill (SYP) is one of the few multi-herb prescriptions of Zhongjing Zhang that is typically prepared for long-term administration. Its effect is to support righteousness by nourishing qi and blood, and it can treat symptoms including fatigue, stomach ache, and irregular periods. SYP, in combination with cisplatin treatment, resulted in more dramatic tumor suppression and significantly reduced mortality in mice with hepatocellular carcinoma, as reported by Zhe Deng et al. (110). Moreover, HIF-1α, PD-1, and PD-L1 expression levels in tumor tissues decreased significantly. Although SYP alone could up-regulate CD4+ T cells, the combination treatment group showed increased CD4+ and CD8+ T cells, demonstrating the synergistic impact of mixing Chinese and Western medicine in T cell detection.

Yuqing Xie’s team (111) investigated the therapeutic role of Yangyin Fuzheng Jiedu’s prescription (YFJP) in hepatocellular carcinoma. They found that Fuzheng prescription (FZP), one of the three dismantled formulas of YFJP, was the most effective in inhibiting tumor growth, with a TGI of 63.1%. It significantly increased thymus and spleen indexes in H22 tumor-bearing mice and the levels of CD8+ and CD3+ T cells in the spleen and peripheral blood. In TME, FZP promoted tumor cell apoptosis, increased tumor-infiltrating CD8+ T cells, and significantly inhibited the inhibitory receptors PD-1, Tim-3, and TIGIT on the membrane. In addition, it reduced IL-1β, IL-4, IL-6, IL-10, and IL-13 levels, down-regulated PI3K, ERK1/2, and up-regulated EGFR levels, improving the anti-tumor effects of the tumor suppressor microenvironment.

Yu Zhang et al. studied the therapeutic facilitation of a herbal in-hospital formula known as CFF-1 for metastatic castration-resistant prostate cancer (112). It was discovered that adding CFF-1 to bicalutamide, goserelin acetate, and docetaxel, the Western primary therapy, can lower patients’ levels of prostate-specific antigen and lessen fatigue, acting as an anti-cancer and quality-of-life enhancement effect. Additionally, studies on animals demonstrated that CFF-1 might block the JAK1/STAT3 signaling pathway to reduce PD-L1 expression and hence have an anti-tumor impact.

## Physiotherapy Sensitizes tumor immunotherapy

### Moxibustion

Moxibustion is a traditional Chinese medicine treatment that involves placing a small amount of burning mugwort on the skin, either a specific area or an acupuncture point, to produce a thermal effect to treat the disease and improve the flow of qi in the meridians and blood circulation. A study found that moxibustion combined with cyclophosphamide chemotherapy effectively inhibited tumor growth in mice with hepatocellular cancer (113). Another study showed that moxibustion treatment, which caused tumor cell necrosis, combined with paclitaxel, significantly reduced tumor growth, improved weight loss, and prolonged survival in breast cancer mice (114). It can also repair the decrease in the thymus index caused by paclitaxel and improve the immune response by increasing IFN-γ and IL-2 and decreasing IL-10 and TGF-β1. Additionally, it inhibited tumor neovascularization through down-regulation of the HIF-1α-VEGF pathway and facilitated immune checkpoint inhibition by down-regulating PD-1 and PD-L1 expression in tumor tissues. Although moxibustion-assisted cancer immunotherapy has not been investigated as part of any clinical trials, it does enhance the quality of life for cancer patients. It has been demonstrated to reduce cancer-related fatigue (115), and Chunhui Wang et al. (116) discovered that moxibustion effectively reduces female breast cancer-related lymphedema.

### Catgut implantation at Acupoint

The catgut implantation at acupoint (CIAA) method, which uses continuous stimulation of acupuncture points to enhance metabolism, improve qi and blood circulation, and relieve symptoms, has been widely used for conditions such as obesity (117, 118) and type 2 diabetes (119). A study by ShiHua Xu et al. (120) found that CIAA reduced the mortality of mice with hepatocellular carcinoma and reduced hepatic histopathological damage. It also decreased the expression of AFP and p-AKT and improved the immune environment by down-regulating PD-1, CTLA-4, and IL-10. Although the study did not include positive controls, it provides valuable insight into the potential benefits of CIAA in cancer immunotherapy. However, more research is needed to fully understand the sensitizing impact of CIAA on tumor immunity.

## Summary and prospects

The down-regulation of PD-1 and PD-L1 prevents their binding and reduces the misidentification of T cells, thereby reducing the immune escape of tumor cells. This is the underlying principle behind ICIs therapy. However, some patients do not respond to these treatments due to tumor heterogeneity and individual drug resistance. Finding drugs that can sensitize cancer immunotherapy, reduce toxicity, and enhance immune function is a crucial challenge in medical oncology. To address this issue, the combination therapy approach should consider sensitization from a sophisticated, coordinated molecular perspective. With this in mind, a review of the PD-1/PD-L1 axis and its regulatory mechanisms was conducted, and it was found that several cytokines and cross-talk pathways can impact its regulation.

**Figure 2:**
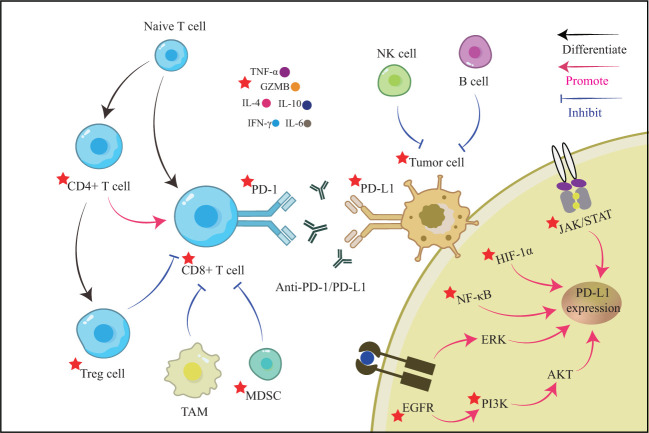
The illustrates TCM sensitization sites in the tumor microenvironment.

We hypothesize that TCM can overcome the limitations of ICIs therapy by serving as an adjuvant to sensitize and reduce toxicity. This hypothesis is based on our observation of existing cancer treatments in China. A thorough review of TCM treatment options, including Chinese medicine monomers, herbal extracts, Chinese herbal formulas, and physiotherapy, was conducted. The findings indicate that TCM can sensitize cancer immunotherapy in multiple ways, such as directly inhibiting the PD-1/PD-L1 axis, regulating T cell function, suppressing immunosuppressive cells, modulating cross-talk signaling pathways, improving the tumor microenvironment, regulating intestinal flora, and more (**Table 1**; **Figure 2**). When combined with PD-1 or PD-L1 mAb treatment, TCM has shown promising synergistic benefits in suppressing tumor volume, increasing longevity, and reducing mortality in tumor-bearing mice.

**Table 1:** TCM therapies sensitize cancer immunotherapy.

Treatment	Disease	PD-1/PD-L1 axis	Other sensitization methods	Cytokines & Molecules	Immune cells	Reference
Ginseng polysaccharides	NSCLC	\	1. Modulate the TME 2. Alter the gut microbiota 3. Influence tryptophan metabolism	IFN-γ↑, TNF-α↑, GZMB↑, IDO↓, L- tryptophan↑, L- kynurenine↓	CD8+ T cell ↑, Treg cell ↓, Th17 cells↓	Jumin Huang (62)
Polysaccharide of Atractylodis Macrocephalae Rhizoma	Esophageal Cancer	PD-L1↓	Regulate microRNAs	miR-34a↑	\	Yicun Han (65)
Honey-processed Astragalus polysaccharides	Melanoma	\	1. Induce tumor cell apoptosis 2. Modulate the TME	\	CD4+T cell ↓, CD8+T cell ↑	Xinrui Sha (70)
Evodiamine	NSCLC	PD-L1↓	1. Induce tumor cell apoptosis 2. Enhance T cell function 3. Modulate the TME	IFN-γ↑, TNF-α↑, GZMB↑, MUC1-C↓	CD8+ T cells↑, Treg cell↓	Zebo Jiang (75)
Oxymatrine	CRC	PD-L1↓	Modulate DNA demethylation	\	\	S.Hua (78)
Gallic acid	CRC	PD-1↓, PD-L1↓	1. Promot Treg fragility 2. Modulate the TME 3. Improve T-cell exhaustion	IFN-γ↑, TNF-α↑, STAT3↓	Treg cells↓	Biaolong Deng (80)
Curcumin	HNSCC	PD-1↓, PD-L1↓, PD-L2↓	1. Modulate the TME 2. Enhance T cell function 3. Suppress epithelial-mesenchymal transition	IFN-γ↑, GZMB↑	CD4+ T cells↑, CD8+ T cells ↑, Treg cells↓	Lihua Liu (84)
Neobavaisoflavone	Breast cancer	\	1. Modulate the TME 2. Promote peripheral immunity	IFN-γ↑, GZMB↑, STAT3↓, Ki-67↑, ROS↓, perforin↑, arginase-1↓	MDSCs↓, CD4+ T cells↑, CD8+ T cells ↑, memory CD4+ T cells↑, memory CD8+ T cells↑,	Jufeng Guo (87)
Quercetin	Breast cancer	PD-1↓, PD-L1↓,	1. Modulate the TME 2. Strengthen PBMC	IFN-γ↑, GZMB↑, IL-2↑	CD8+ T cells ↑	Lei Jing (90)
Andrographolide	NSCLC	PD-L1↓	1. Induce tumor autophagy 2. Modulate the TME 3. Inhibit JAK/STAT pathway	IFN-γ↑, TNF-α↑, GZMB↑, STAT3↓, Ki67↓, ROS↑	CD8+ T cells ↑	Xuanrun Wang (91)
Sesquiterpene lactone-rich fraction of *Inula helenium* L.	CRC	\	1. Modulate the TME 2. Promote T-cell infiltration	GZMB↑, TGF-β1↓	CD8+ T cells ↑, M1-like macrophages↑	Jaemoo Chun (92)
Platycodon grandiflorum	NSCLC	PD-1↓	1. Modulate the TME 2. Inhibit the VEGF pathway	IFN-γ↑, GZMB↑, STAT3↓, VEGF-A↓, IL-6↓, IL-10↓	CD8+ T cell ↑	Ruijie Yang (96)
Aqueous extract of Taxus chinensis var. mairei	NSCLC	PD-1↓	1. Restore macrophage capacity 2. Modulate the TME	CD47↓	Treg cell ↓	Shuying Dai (98)
Gegen Qinlian decoction	CRC	PD-1↓	1. Modulate the TME 2. Alter the gut microbiota	IFN-γ↑, IL-2↑	CD8+T cell↑	Ji Lv (101)
Dahuang Fuzi Baijiang decoction	CRC	PD-1↓	1. Alleviate T-cell exhaustion 2. Modulate the TME	IL-6↓, CCL2↓	CD8+T cell↑	Yihua Xu (102)
Qingfei Jiedu decoction	NSCLC	PD-L1↓	1. Inhibit EGFR, HIF-1, NF-κB pathway 2. Promote MAPK/AKT pathway	AKT1↑, MAPK1↑, EGFR↓, HIF1A↓, JUN↓, RELA↓, NFKBIA↓	CD8+T cell↑	Junjie Pan (103)
Modified Buzhong Yiqi decoction	Gastric Cancer	PD-L1↓, PD-1↓	Modulate the TME	IFN-γ↑, PI3K↓, AKT↓	CD4+/CD8+ T cells ↑, CD8+ T cell↓, Treg cells↓	Ruihan Xu (109)
Shuyu pills	HCC	PD-1↓, PD-L1↓	1. Modulate the TME 2. Inhibit the HIF-1 pathway	HIF-1α↓	CD4+T cell↑, CD8+T cell↑	Zhe Deng (110)
Fuzheng prescription	HCC	PD-1↓	1. Alleviate T-cell exhaustion 2. Modulate the TME 3. Inhibit the PI3K pathway	PI3K↓, EGFR↑, ERK1/2↓, IL-1β↓, IL-4↓, IL-6↓, IL-10↓, IL-13↓	CD8+T cell↑	Yuqing Xie (111)
CFF-1	prostate cancer	PD-L1↓	1. Modulate the TME 2. Inhibit the JAK1/STAT3 pathway	JAK1↓, STAT3↓	\	Yu Zhang (112)
Moxibustion	Breast cancer	PD-1↓, PD-L1↓	1. Modulate the TME 2. Suppress tumor neovascularization	IFN-γ↑, TGF-β↓, IL-2↑, IL-10↓, HIF-1α↓, VEGFA↓	\	Ning Xue (114)
Catgut Implantation at Acupoint	HCC	PD-1↓	1. Modulate the TME 2. Inhibit the AKT pathway	AFP↓, p-AKT↓, CTLA-4↓, IL-10↓	\	Shihua Xu (120)

NSCLC, Non-small cell lung cancer; CRC, Colorectal Cancer; HNSCC, Head and neck squamous cell carcinoma; HCC, Hepatocellular carcinoma; TME, Tumor microenvironment; PBMC, Peripheral blood mononuclear cell; IFN-γ, Interferon-γ; TNF-α, Tumor necrosis factor-α; GZMB, granzyme B; IDO, Indoleamine -2, 3-dioxygenase; ROS, Reactive oxygen species; PI3K, Phosphatidylinositol-3-kinase; STAT3, Signal Transducer And Activator Of Transcription 3; IL-2, Interleukin-2; IRF1, Interferon Regulatory Factor 1; MAPK1, Mitogen-activated protein kinase 1; EGFR, Epidermal growth factor receptor; HIF1A, Hypoxia inducible factor 1; CTLA-4, Cytotoxic T lymphocyte–associated antigen 4.

“↑” means the expression of this indicator is upregulated.

“↓” means the expression of this indicator is downregulated.

“\” means that this literature does not contain such indicators.

Advancements in technology, such as fingerprinting, have led to a standardization of the quality of Chinese medicines (121). The potent tumor-killing and immune-boosting effects of various herbal compounds have been acknowledged. However, Chinese herbal formula use is still limited by factors like the concentration of the compounds and a lack of clarity in their pharmacodynamic ingredients. New technologies such as microfluidic microarrays, organoid microarrays, and artificial intelligence are being developed to identify the critical efficacy molecules to address this issue. Additionally, the growth and advancement of herb genomics research and the formation of a comprehensive Chinese medicine database are helping to create a complete mechanism for understanding and further clinical application and industrial development of Chinese medicine (122).

In conclusion, TCM can enhance cancer immunotherapy by sensitizing immune checkpoints through various mechanisms, making combination therapy a potentially more beneficial option for oncology patients. Further research is crucial in exploring the role of TCM in cancer immunotherapy, as it holds the promise of boosting immune response and overcoming the limitations of ICIs treatments.

## Author contributions

All authors listed have made a substantial, direct, and intellectual contribution to the work and approved it for publication.
